# Biologically Inspired Hierarchical Contour Detection with Surround Modulation and Neural Connection

**DOI:** 10.3390/s18082559

**Published:** 2018-08-04

**Authors:** Shuai Li, Yuelei Xu, Wei Cong, Shiping Ma, Mingming Zhu, Min Qi

**Affiliations:** 1 Aeronautical Engineering College, Air Force Engineering University, Xi’an 710038, China; lishuailisuai@163.com (S.L.); kgy_congwei@163.com (W.C.); mashiping@126.com (S.M.); ming_paper@163.com (M.Z.); 2 Unmanned System Research Institute, Northwestern Polytechnical University, Xi’an 710072, China; 3 School of Electronics and Information, Northwestern Polytechnical University, Xi’an 710072, China; drqimin@nwpu.edu.cn

**Keywords:** contour detection, hierarchical biological vision, surround modulation, receptive field, neural connection

## Abstract

Contour is a very important feature in biological visual cognition and has been extensively investigated as a fundamental vision problem. In connection with the limitations of conventional models in detecting image contours in complex scenes, a hierarchical image contour extraction method is proposed based on the biological vision mechanism that draws on the perceptual characteristics of the early vision for features such as edges, shapes, and colours. By simulating the information processing mechanisms of the cells’ receptive fields in the early stages of the biological visual system, we put forward a computational model that combines feedforward, lateral, and feedback neural connections to decode and obtain the image contours. Our model simulations and their results show that the established hierarchical contour detection model can adequately fit the characteristics of the biological experiment, quickly and effectively detect the salient contours in complex scenes, and better suppress the unwanted textures.

## 1. Introduction

Contour detection refers to the identification of the boundary between the target and the background in an image, focusing on the dividing lines outside the targets [1]. As an important mid-level visual task, contour detection can greatly reduce the image dimension while retaining important structures and rejecting a large amount of weakly related information, which lays an important foundation for advanced computer vision tasks (such as target interpretation [2] and image analysis [3]).

A great deal of the work in early studies tried to extend conventional edge detection methods (such as Canny [4] and Normalised Cuts [5], etc.) to contour detection; however, in contrast to the primary edge detection task, the essence of contour detection is complete separation of the foreground and background or each area of an image. Detailed information, such as texture and structure, within the target or area must be selectively suppressed, thereby highlighting the demarcation of the subject in the foreground or between areas. In recent years, to solve the problem of contour extraction in complex scenes, many new methods based on supervised machine learning have been successively proposed [6,7,8,9]. Martin et al. [6] proposed the Pb method by defining gradient operators for brightness, colour, and texture channels, using them as input to a logistic regression classifier for predicting edge strength, and finally integrating the predicted edges with supervised learning method. In subsequent studies, researchers further improved the effect of contour detection mainly by introducing technologies, such as multi-feature [10], multi-scale [11] and global information [12]. However, this supervised method relies too much on the training sets and has low computational efficiency. The human visual system (HVS) has powerful visual information processing capabilities, which is the key to the survival of the vast majority of living organisms [13]. Brain-like computing can help us quickly and accurately process the information received, and it is the core of achieving artificial intelligence. A large amount of biological experimental data show that cells at the early vision stages (for example, lateral geniculate nucleus (LGN), primary visual cortex (V1) and secondary visual cortex (V2) et al.) can not only extract motion information of the moving targets [2] but also encode colour, edge, and azimuth information [14,15,16]. In this paper, consideration is given to drawing from the contour detection capabilities of the nerve cells to establish a hierarchical computational model under the inspiration of biological vision to quickly and effectively extract the contours of natural images and lay a foundation for subsequent in-depth research.

Since the mid-20th century, many researchers have focused on the field of computer vision and established a great variety of extraction models for features such as texture and contour. The orientation selectivity of the receptive fields is the theoretical basis of the contour detection algorithms based on the biological vision mechanisms. In 1965, Rodieck et al. [17] proposed the use of the difference-of-Gaussian (DOG) model for carrying out experiments on the concentric antagonistic structures of the retina and the LGN-cell classical receptive field (CRF). Daugman et al. [18] then proposed that a two-dimensional Gabor filter can well mimic the receptive field characteristics of V1 simple cells and obtain the local edge intensity of images. Detecting image contours from a complex natural scene requires the suppression of cluttered textures within the target or the area, thereby highlighting the contour lines. However, due to the complex factors in natural scenes, such as noise, light, colour, and texture, edge detection methods cannot guarantee the capture of closed and complete contour information. Additionally, existing texture suppression methods cannot measure up to the capabilities possessed by biological vision and are often limited to a certain aspect or field. Subsequently, neurophysiological and neuropsychological studies on monkeys and cats have clearly demonstrated that there is a peripheral region beyond the CRF, known as non-classical receptive field (NCRF), can mutual modulate the spiking response of a neural cell at early vision stages on to the stimuli placed within the CRF—that is contextual influence or center-surround interaction—which highlights the texture of the isolated edge group, achieving the reduction of background textures while better highlighting the real boundaries of the target area [19]. In 2003, Grigorescu et al. [20] propose a contour detector for grayscale images—that is CORF model—which aims to improve the performance of classical edge detectors by trying to inhibit the response of the gradient operator on textured regions of input images. Papari et al. [21] propose a biologically inspired multiresolution contour detector by using Bayesian denoising together with the NCRF surround inhibition.

Although all above methods preliminarily integrate the HVS with the computer vision system, especially by considering the models and mechanisms of biological vision to directly obtain the image features, the fit of the perceptual characteristics of visual cells for features such as edges, shapes, and colours is still inadequate, and the role of V2 cells on extended lines and textures in addition to sharing many properties of V1 cells is also rarely applied to contour detection [22,23]. In addition, studies in neuroscience have shown that the feedforward, lateral, and feedback neural connections between the cells at the same layer and between layers play a key integration role in information expression and better suppress the unwanted edges in the texture regions [24]. Thus, by simulating the information processing mechanisms for the receptive fields of the cells in the early visual system, we propose a biologically inspired hierarchical contour detection (BIHCD) model to quickly and effectively detect the region boundaries in complex scenes. This paper is focused on studying the receptive fields and functional characteristics of the cells at early visual stages in combination with existing neuron models and the visual perception mechanisms, and on proposing a connection platform for the HVS and the computer vision system with the goal of detecting salient contours.

The remaining of this paper is organized as follows: in Section 2, we present a small representative and relevant subset of related works. In Section 3, we briefly give the general framework of our BIHCD model and describe the details of image feature extraction with CRF models of the cells at early visual stages, the NCRF surround modulation with multifeatured-based weight combination inhibition, and the full multi-scale guided contour detection model. In Section 4, we evaluate and analyze the performance of our model on complex natural scenes, such as BSDS300/500 datasets. Finally, in Section 5 we draw the conclusions.

## 2. Related Works

Contour detection is a classical and important problem in intelligent computation, image processing and computer vision. After large numbers of researchers has done a lot of works, the contour detecting technology has made great developments at multi-directions, and enormous state-of-art algorithms on this topic are proposed. It is hard to give a full survey on contour detection problem, so we only review a small representative and relevant subset of literatures in this paper.

### 2.1. Contour Detection

The early contour detection studies relied on local and low-level features with differential operator and mathematical morphology, such as Canny [4], Sobel [25], Prewitt [26] and Normalized Cuts [5], etc. All of these methods use local filters of a fixed scale and few features. Many unwanted edges and cluttered backgrounds are treated equally as meaningful contours in these detectors. Typically, Dollar et al. [7] proposed an approach with rich patch features and probabilistic boosting tree, which achieved real-time contour detection. However, these learning-based methods are inflexible for “untrained” images because their performances are strongly dependent on the completeness of training datasets. Therefore, many non-learning-based methods have been proposed recently. Arbelaez et al. [1] combine multiple local cues (i.e., local Pb measure [6]) into a spectral clustering framework through supervised learning method for contour detection. Isola et al. [27] measured rarity based on pointwise mutual information. However, the computation cost is also exhausted.

### 2.2. Biologically-Inspired Methods

Because of the excellent performance of HVS on image processing, a number of biologically-inspired contour detection algorithms have been proposed with promising results by now. ViCBiC team of UESTC has done a lot of works on contour detection [28,29,30,31,32]. For example, a butterfly-shaped and orientation-selective model is proposed to reduce responses in homogeneous region and maintain responses in inhomogeneous region with the inhibiting weights by orientation difference of CRF and NCRF of V1 neurons [28]. Zeng et al. dramatically removed the non-meaningful texture elements by introducing an adaptive two-scale mechanism into the model [29]. Further, Yang et al. explored imbalanced colour opponency [30] and the spatial sparseness constraint in V1 [31] to reserve the desired fine boundaries. Besides of their works, Spratling et al. [33] proposed a predictive coding and biased competition mechanism to model the sparsity coding of neurons in V1. Wei et al. [34] presented a butterfly-shaped inhibition model operating at multiple spatial scales. Feedback projections for top-down modulation have been studied and modelled in computer vision proposals [35,36,37]. More recently, feedback connection has also been considered on contour detection. D´ıaz-Pernas et al. [35] extracted edges through oriented Gabor filters accompanied with top-down and region enhancement feedback layers. Neumann et al. [36] proposed a new recurrent model for the interaction between V1–V2, including elongated receptive fields for texture processing. Raudies et al. [37] proposed feedback interactions from MT to V1 to enhance the motion signals.

In conclusion, much remains to be investigated about the functional role and the underlying mechanisms of surround inhibition and connections in neurons in contour detection of natural scenes. The bio-inspired models above only mimic part of the visual processing mechanisms in HVS. In this paper, we are especially concerned with incorporating recent knowledges of the physiological properties of cortical neurons. We will try to construct better solution to the color-texture suppression and contour integration by putting forward a distance-weighted colour-oriented encoding approach with multifeature-based surround modulation. Furthermore, we will explore the neural connections and pooling mechanism between visual areas with new physiological findings.

## 3. The *BIHCD* Model

**Figure 1:**
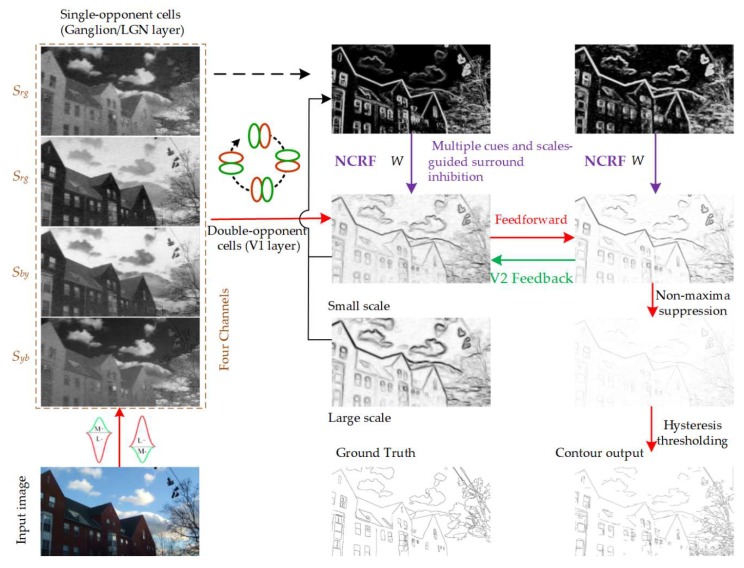
Flowchart of our BIHCD model framework.

The general flowchart of the contour detection algorithm proposed in this paper is summarized in Figure 1. The proposed model mimicking the human visual processing stages is a hierarchical visual architecture. According to differentiation between anatomical structure and physiological function, the frontal cortex or brain areas responsible for contour and boundary detection in the HVS include the Retina, LGN, V1, and V2, etc. First, the input image is perceived and captured by single-opponent ganglion cells and LGN cells, which also carry out a convolution operation to form an image with four paths of different colours for the opponent channels; it is then further processed by the double-opponent V1 cells with different orientation sensitivities to preliminarily obtain the local edges of colour and luminance for the image. Next, to better solve the problems of texture suppression and contour integration in complex natural scenes, we use a multi-feature suppression and integration method under the guidance of multi-scale information and put forward a distance-weighted colour-oriented encoding method that automatically modulates the suppression strength of each pixel position using the surround inhibition weight obtained after integration. Finally, the feedforward and feedback processing of the V2 in response to the edges and textures of the cells in V1 is introduced, and methods such as non-maxima suppression and hysteresis threshold are used for processing the contours, such as refinement and integration, to obtain the hierarchical expression of the final image contours.

### 3.1. Classical Receptive Field Models for Hierarchical Contour Detection

#### 3.1.1. LGN

After processing the visual signals, the retinal ganglion cells transfer the feedforward to the LGN. Although the receptive field of the LGN cells is larger than that of the ganglion cells, the characteristics of the receptive fields for these two types of nerve cells are basically consistent. Therefore, a great deal of research works [10,23,31] merge the two for studying and view them as one layer. The receptive field of cells in this layer has the single-opponent and spatially low-pass properties. Biological experimental studies have shown that the visual system transfers visual information such as image colours through the two opponent channels of red-green (*r-g*) and blue-yellow (*b-y*). The single-opponent characteristic can be used to segment the different contour areas in the image through their luminance and colour information. The cells in this layer receive the retina outputs, and their responses can be described by:

(1) $$\begin{array}{l} S_{rg}\left(x,y,\sigma \right)=\pm (I_{r}(x,y)*G\left(x,y,\sigma \right)+w\cdot I_{g}(x,y)*G\left(x,y,\sigma \right))\\ S_{by}\left(x,y,\sigma \right)=\pm (I_{b}(x,y)*G\left(x,y,\sigma \right)+w\cdot I_{y}(x,y)*G\left(x,y,\sigma \right)) \end{array}$$

where *I* (*I_r_*, *I_g_*, *I_b_*) is the input image, the yellow components *I_y_* = (*I_r_ + I_g_*)/2; *w* represents the weights of the retinal inputs into the ganglion cells (*w* ∈ [−1.0, 0]); * represents the convolution operation; and in order to obtain the local information, the Gaussian smoothing filters (i.e., *G*(∙)) are used to represent the response generated by the nerve cells to the visual stimuli of a specific area. We use Gaussian filters with same standard deviate (*σ*) to ensure that each channel has the same size of receptive field. *S_rg_*(+) and *S_rg_*(−) respectively represent the responses of two types of cells, *r-on/g-off* and *r-off/g-on*; similarly, we can get the responses of *b-on/y-off* and *b-off/y-on* cells (i.e., *S_by_*(+) and *S_by_*(−)), and its physiological significance is in enabling the visual system to flexibly detect the contour lines formed by the black and white boundaries and colour contrast boundaries.

#### 3.1.2. V1

The nerve cells in the cortex layer of V1 are the key links in visual information processing. They are generally divided into two types: simple cells and complex cells, which can effectively encode static information such as colours, edges, and structures. The simple-cell receptive field shows a slender and long shape, is comparatively sensitive to striation stimuli, and can detect simple shapes such as lines and edges [38]. The cells’ receptive field that processes colour information has a double-opponent characteristic for colour and space at the same time. On the other hand, the complex cell receptive field can be formed by superimposing the simple-cell receptive fields [39]. In this paper, the real needs of contour detection are combined with comparatively low computational complexity. In connection with the receptive field characteristics of the V1 cells, the first derivative of the Gaussian function with variance *σ* is selected for simulating the establishment of the spatial structure for the simple-cell receptive field, as shown in Equations (2) and (3):

(2) $$\begin{array}{l} f\left(x,y,\theta ,\sigma \right)=\frac{1}{2\pi {\left(k\sigma \right)}^{2}}\exp(-\frac{{\tilde{x}}^{2}+{\left(\lambda \tilde{y}\right)}^{2}}{2{\left(k\sigma \right)}^{2}})\\ {\left[\tilde{x}\;\tilde{y}\right]}^{T}={\left[x\cos(\theta )+y\sin(\theta )-x\sin(\theta )+y\cos(\theta )\right]}^{T} \end{array}$$

(3) $$V\left(x,y,\theta ,\sigma \right)=\frac{\partial f(x,y,\theta ,\sigma )}{\partial \tilde{x}}=-\frac{\tilde{x}}{k^{2}\sigma^{2}}\cdot f(x,y,\theta ,\sigma )$$

where *λ* represents the length-to-width ratio of the receptive field space, *θ* is the preferred direction of a given V1 cell, *k* determines the contrast in the spatial size of the V1-cell receptive field and the LGN-cell receptive field, and *σ* parameterizes the RF size (same as the scale of Gaussian filters used in the ganglion/LGN layer); therefore, *λ* = 0.5, *σ* = 1.1, *k* = 2, *θ* ∈ [0, 2π] is set up in this paper based on [19,31].

At the same time, input to the two sub-areas of the simple-cell double-opponent receptive field receives the output of LGN single-opponent nerve cells with two opposite polarities, respectively; therefore, the V1-simple-cell receptive field response is given by:

(4) $$E_{rg}\left(x,y,\theta_{i},\sigma \right)=S_{rg}\left(+\right)*\left\lfloor V\right\rfloor +S_{rg}\left(-\right)*\left\lfloor -V\right\rfloor$$

since the cell response can be only a non-negative value in accordance with the basis of the biological theory, the method of half-wave rectification is used in processing; that is, ⌊V⌋=max{0,V}. In addition, *θ_i_* represents the *i*-th preferred direction of a given set of simple cells (here, *θ_i_*= (*i* − 1)2π/*N*, *i* = 1, 2, …, *N*), and *N* = 6 is set up in this paper. This set of double-opponent receptive field models can be used to detect the image boundaries defined by different luminance and color.

The response of the V1 complex cells can be obtained by integrating the output of the simple cells. At present, there are three main integration models for the simulation of complex cells: the *MAX* model, which takes the maximum value; the energy model, which seeks the sum of squares; and the learning model with data training. To maintain better edge extraction characteristics, the *MAX* model is selected in this paper; that is, the V1 complex cell output of this channel is defined by the maximal value of the response of simple cells with *N* orientations from this given group, as shown in Equation (6):

(5) $${\tilde{E}}_{rg}\left(x,y,\sigma \right)=\max\{E_{rg}\left(x,y,\theta_{i},\sigma \right)|i=1,2,\ldots ,N\}$$

To improve the capabilities of the V1 cells in perceiving direction and, at the same time, avoid the ‘white wall’ problem [40]—that is, divisions by zero in regions with no energy (when no spatio-temporal texture is present), we use the tuned and untuned normalisation proposed in [41], which combines with a static, non-linear self-normalisation over maximum edge responses at each spatial position. So, the final responses of V1 complex cells is defined by

(6) $$D_{rg}\left(x,y,\sigma \right)=\frac{{\tilde{E}}_{rg}^{2}\left(x,y,\sigma \right)}{\max({\tilde{E}}_{rg}^{2}\left(x,y,\sigma \right))+\varepsilon }$$

where *ε* is an extremely small positive constant.

**Figure 2:**
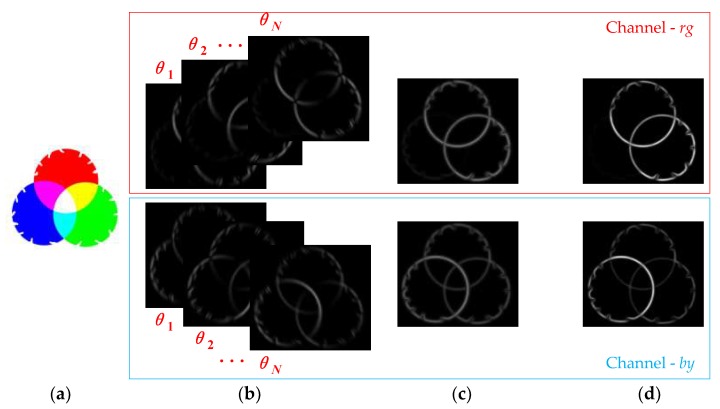
Representation of the V1 RFs’ responses. (**a**) Input, (**b**) V1-simple-cell response, (**c**) MAX integration, (**d**) V1-complex-cell response.

The normalisation operation can strengthen the wanted edges while weakening the internal texture information. Figure 2 represents the V1 RFs’ responses at each stage. In the same way, the responses of the other three colour opponent channels—*D_gr_*(*r-off/g-on*), *D_by_* (*b-on/y-off*), and *D_yb_* (*b-off/y-on*)—can be obtained through the above equations.

#### 3.1.3. V2

In 2016, Pu’s team used a combination of optical imaging information and an electro-physiological method to obtain a V2 neuron receptive field model [42], from which the high-resolution spatial structure of the V2 receptive field was obtained and divided the receptive fields of the V2 nerve cells into three categories: a type similar to the V1-cell receptive field; slender type; and complex structure type (containing multi-oriented components). The structures of the receptive field for these V2 cells can be explained by the integration of the V1-cell receptive field. Based on this major physiological finding, we comprehensively considered selecting the elliptical Gaussian function along the optimal direction [43] to fit the characteristics of the V2-cell receptive field according to:

(7) $$V_{2-rg}\left(x,y,\sigma \right)=D_{rg}\left(x,y,\sigma \right)*f_{ellipse}(\sigma \prime ,\theta_{⊥})$$

Here, $f_{ellipse}(\cdot )$ is the elliptical Gaussian function along the optimal direction, defined as:

(8) $$f_{ellipse}(x,\;y)=e^{-(ax^{2}-2bxy+cy^{2})}$$

where $a=\frac{\cos^{2}\theta }{2\sigma^{2}}+\frac{\sin^{2}\theta }{2\sigma \prime^{2}},\;b=-\frac{\sin2\theta }{4\sigma^{2}}+\frac{\sin2\theta }{4\sigma \prime^{2}},\;\mathrm{and}\;c=\frac{\sin^{2}\theta }{2\sigma^{2}}\cdot \frac{\cos^{2}\theta }{2\sigma \prime^{2}}$.

The visual information transfer process is an information processing flow with a basis in the hierarchical structure of the visual perception system. In addition to the aforementioned feedforward and lateral connections, there is a large amount of feedback information transfer in the visual information flow between the cells of various levels and areas. Therefore, in combination with the contents of the study, we considered only the edge information feedback from V2 to V1 in this work corresponding to the well established fact that global shape influences local contours [32,34,35,36]. By maximizing the responses of V2 cells in all four channels (i.e., *rg*, *gr*, *yb*, *by*), we simulated the global shape and sent it as feedback to V1. This feedback is processed only one time same as all other inputs to V1. The final edge response of our model is obtained according to:

(9) $$D\left(x,y\right)=\underset{feedforward}{\underset{︸}{\sum_{channel}V_{2-channel}}}+\underset{feedback}{\underset{︸}{feedback(\underset{channel}{\max}\{V_{2-channel}\})}},\;\mathrm{with}\;channel\in \left\{rg,gr,yb,by\right\}$$

Obviously, *D*(*x*, *y*) is the final edge map, which includes two parts: the basic feedforward information and the feedback enhancement.

### 3.2. Surround Modulation Method of the NCRF

In general, the peripheral area of the aforementioned CRF has a certain surround inhibition effect on the response of the central receptive field, thereby highlighting the texture of isolated edge groups and achieving the reduction of background texture edges while better highlighting the boundaries of the target region. The NCRF introduces an important viewpoint concerning the local environmental background, and the environment can have specific features. It also enlarges the receptive field of the nerve cells by multiple folds, providing an affective neural mechanism for the visual system to perceive image features under complex natural scenes. It is exactly the interactions based on the complex nonlinearity between CRF and NCRF that lead to the diversity of cell response characteristics, and when there is texture in the environment, the NCRF will generate an obvious suppression modulation effect on the CRF response, thereby reducing the response of unwanted textures in the cluttered background to highlight the boundaries of the contour region. This kind of suppression modulation effect depends on the degree of difference in features such as luminance, distance, direction, and colour between the centre of the receptive field and the surrounding environment. Here, a variety of local features that affect the texture suppression modulation effect are extracted. First, we adopt the distance-related weighting function as:

(10) $$W_{d}(x,y)=DOG_{\sigma ,\rho }^{+}\left(x,y\right)/{\left\|DOG_{\sigma ,\rho }^{+}\left(x,y\right)\right\|}_{1}$$

where ${\left\|\cdot \right\|}_{1}$ denotes the *L*_1_ norm, and $DOG_{\sigma ,\rho }^{+}\left(x,y\right)$ is calculated as:

(11) $$DOG_{\sigma ,\rho }^{+}\left(x,y\right)=\left\lfloor \exp(-\frac{\left(x^{2}+y^{2}\right)}{2{\left(\rho \sigma \right)}^{2}})\frac{1}{2\pi {\left(\rho \sigma \right)}^{2}}-\exp(-\frac{\left(x^{2}+y^{2}\right)}{2\sigma^{2}})\frac{1}{2\pi \sigma^{2}}\right\rfloor$$

According to electrophysiological experiments, the size of the NCRF is normally two to five times (in diameter) that of the CRF [44]. In this work we set *ρ* = 0.4.

On the other hand, surround inhibition strength decreases also with the increasing feature difference between the CRF and non-CRF. In this paper, we evaluate the contribution of local luminance and luminance contrast in contour detection with surround inhibition. We calculate the luminance and luminance contrast in local patches formed by a raised sine-weighted window:

(12) $$w\left(x_{i},y_{i}\right)=(\cos(\pi \sqrt{{\left(x_{i}-x\right)}^{2}+{\left(y_{i}-y\right)}^{2}})/\delta +1)/2$$

where *δ* is the radius of the local window *B_xy_*, and (*x_i_*, *y_i_*) represents the *i*-th pixel of the image window with the centre at (*x*, *y*). Here, we set *δ* = 0.5; then, *B_xy_* is the sliding window that contains 11 × 11 pixels. Therefore, the local luminance and luminance contrast features can be obtained according to:

(13) $$L\left(x,y\right)=\frac{1}{\eta }I\left(x,y\right)*w(x,y)$$

(14) $$C\left(x,y\right)=\sqrt{\frac{1}{\eta }({\left(I\left(x,y\right)-L\left(x,y\right)\right)}^{2})/(L{\left(x,y\right)}^{2})*w(x,y)}$$

where $I\left(x_{i},y_{i}\right)=0.299I_{r}\left(x_{i},y_{i}\right)+0.587I_{g}\left(x_{i},y_{i}\right)+0.114I_{b}\left(x_{i},y_{i}\right)$, $\eta =\sum_{\left(x_{i},y_{i}\right)\in B_{xy}}w(x_{i},y_{i})$. To facilitate computation, the prominent contour boundaries are effectively integrated, and the local luminance *L* and the luminance contrast *C* are processed by linear normalisation (*L*, *C* ∈ [0, 1]).

Since the strength of surround inhibition attenuates as the differences in luminance and contrast increase, we define the modulation weight of the NCRF peripheral midpoint (*x_i_*, *y_i_*) on the cells of the CRF centre point (*x*, *y*) as:

(15) $$\begin{array}{l} W_{\Delta L}\left(x,y,x_{i},y_{i}\right)=\exp(-\frac{\Delta L{\left(x,y,x_{i},y_{i}\right)}^{2}}{2\sigma_{1}^{2}})=\exp(-\frac{{\left(L\left(x,y\right)-L\left(x_{i},y_{i}\right)\right)}^{2}}{2\sigma_{1}^{2}})\\ W_{\Delta C}\left(x,y,x_{i},y_{i}\right)=\exp(-\frac{\Delta C{\left(x,y,x_{i},y_{i}\right)}^{2}}{2\sigma_{1}^{2}})=\exp(-\frac{{\left(C\left(x,y\right)-C\left(x_{i},y_{i}\right)\right)}^{2}}{2\sigma_{1}^{2}}) \end{array}$$

where the standard deviation *σ*_1_ determines the rate at which the strength of surround inhibition attenuates as the differences in luminance and contrast increase; we set *σ*_1_ = 0.05 in this work.

At the same time, considering the attenuation of the suppression effect by the luminance and contrast of pixel points at different distances in the periphery on the centre point as distance increases, and finally, under the modulation of luminance and contrast, the suppression weight coefficients of the periphery on the CRF centre at (*x*, *y*) are, respectively:

(16) $$\begin{array}{l} W_{L}\left(x,y,x_{i},y_{i}\right)=\sum_{\left(x_{i},y_{i}\right)\in R_{NCRF}}W_{\Delta L}\left(x,y,x_{i},y_{i}\right)W_{d}\left(x_{i}-x,y_{i}-y\right)\\ W_{C}\left(x,y,x_{i},y_{i}\right)=\sum_{\left(x_{i},y_{i}\right)\in R_{NCRF}}W_{\Delta C}\left(x,y,x_{i},y_{i}\right)W_{d}\left(x_{i}-x,y_{i}-y\right) \end{array}$$

where *R_NCRF_* represents the spatial scope of the NCRF determined by the *DOG*^+^ in Equation (6).

In connection with the distance, direction, and colour features of a given image, we propose integration of the three for processing. This is consistent with the biological characteristics of cells in the visual cortex—that is, the complexity of the surround inhibition effect of the cells. In addition, compared with some existing methods [10,34,36], features such as colour, distance, and direction are fused in this paper, and a distance-weighted colour-oriented encoding method is proposed, which overcomes the noise interference that taking a single extremum from the local area is easily susceptible to and solves the problem of difficulty in detecting the colour boundaries of weak texture (as shown in Figure 2).

To describe the suppression effect of the NCRF periphery on the difference in the colour orientation of the receptive field at the centre of the CRF, the maximal value of the response in each direction of the four opponent channels is taken as the response of that colour orientation—that is:

(17) $$\theta \left(x,y,\theta_{i},\sigma \right)=\max\{{\tilde{E}}_{co}\left(x,y,\theta_{i},\sigma \right)|co\in \{rg,gr,by,yb\}\}$$

As mentioned above, surround inhibition strength decreases also with the increasing feature difference between the CRF and non-CRF. To effectively represent the orientation difference between the texture patterns within CRF and non-CRF, the difference in the colour orientation of the receptive field centre-periphery by further considering the distance-weighted factors is defined as:

(18) $$\begin{array}{l} \Delta \theta \left(x,y,\sigma \right)=\sqrt{{\left\|{\bar{\theta }}_{C}\left(x,y,\sigma \right)-{\bar{\theta }}_{S}\left(x,y,\sigma \right)\right\|}^{2}}\\ =\sqrt{\sum_{i=1}^{N}{\left\|\theta \left(x,y,\theta_{i},\sigma \right)*G(x,y,\delta )-\theta \left(x,y,\theta_{i},\sigma \right)*DOG_{\sigma ,\rho }^{+}\left(x,y\right)\right\|}^{2}} \end{array}$$

where ${\bar{\theta }}_{C}$ denotes the Gaussian-weighted mean vector of the orientation vector θ in the CRF, and ${\bar{\theta }}_{S}$ represents the *DOG*^+^-weighted mean vector of the orientation vector in the NCRF.

Finally, the surround modulation weight of colour orientation selectivity is:

(19) $$W_{\theta }\left(x,y\right)=\exp(-\frac{\Delta \theta {\left(x,y,\sigma \right)}^{2}}{2\sigma_{2}^{2}})$$

where the standard deviation *σ*_2_ determines the rate at which the strength of surround inhibition attenuates as the difference in colour orientation increases; here, we set *σ*_2_ = 0.2.

**Figure 3:**
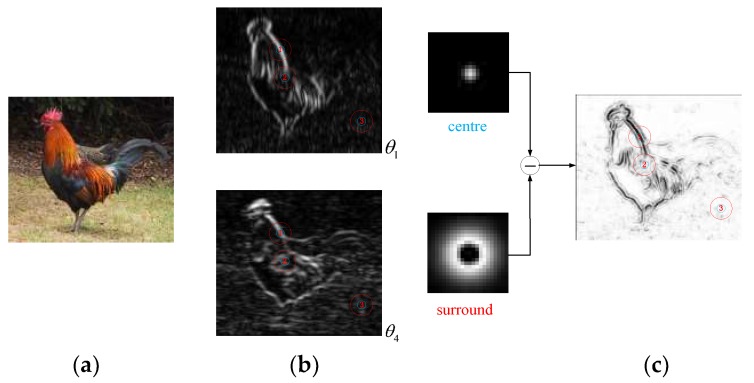
Examples of computing colour orientation difference for typical texture patterns. (**a**) Input, (**b**) Colour orientation responses (*θ*_1_,…,*θ_N_*), (**c**) Surround modulation weights of colour orientation.

Figure 3 shows the examples of computing colour orientation difference Δθ on three types of texture patterns. The colour orientations at point 1 in the center and surround are quite different (high possibility of being contours), which results in a large difference between ${\bar{\theta }}_{C}$ and ${\bar{\theta }}_{S}$, and hence a high Δθ. In contrast, colour orientations in the center and surround are quite similar in the examples shown at point 2 and 3 (low possibility of being contours), which results in a relatively low.

### 3.3. Multi-Scale Guided Contour Extraction

In this paper, we use a multi-feature suppression and integration method under the guidance of multi-scale information (see reference [10] for more details). First, the strength of surround inhibition is integrated according to:

(20) $$W\left(x,y\right)=\{\begin{array}{c} \max(W_{\theta },\;W_{L},\;W_{C})(x,y)\;\Delta D_{CRF}\geq 0\\ \min(W_{\theta },\;W_{L},\;W_{C})(x,y)\;\Delta D_{CRF}<0 \end{array}$$

where Δ*D_CRF_* is the difference in the edge energy of the extracted receptive fields under two scales (*σ* and 2*σ*). Generally, the CRF responses at a large scale (i.e., *D* (*x*, *y*, 2*σ*)) includes reliable contours, but also misses the wanted detailed edges. In contrast, the CRF responses at a fine scale (i.e., *D* (*x*, *y*, *σ*)) covers more details, also including the unwanted edges in texture regions. Therefore, if its value is greater than or equal to zero, this pixel position is an internal texture that must be suppressed; otherwise, it is a contour edge that must be retained. In this work, we set *σ* = 1.1.

Finally, the hierarchical neuronal responses to the contours of the input image can be computed through subtracting the combined weight (i.e., *W* (*x*, *y*)) modulated surround inhibition from the CRF responses at the fine scale according to:

(21) $$CD\left(x,y\right)=\left\lfloor D\left(x,y,\sigma \right)-\rho \cdot W\left(x,y\right)\cdot (D\left(x,y,\sigma \right)*W_{d}\left(x,y\right))\right\rfloor$$

where *ρ* is a texture attenuation factor that denotes the connection weight between the CRF and its surrounding NCRF.

## 4. Experiments and Results

In this section, we first test the edge extraction performance and analyse the basic characteristics of the proposed model on several simple images. Then, we further investigate the response characteristics of the CRF centre and the multi-feature guided texture surround inhibition of the NCRF peripheral with a complex human image. Finally, we give experimental results on a popular natural image dataset (i.e., BSDS300/500) [45], respectively evaluate the performance of our model from subjective and objective perspectives, and analyse the influence of model parameters on the test results.

To compare the algorithms conveniently, we compute the average precision (AP) and the so-called F-measure, which have been widely used for evaluating the performance of edge detectors [6,23]. F-measure signals the similarity between the contours identified by the algorithm and human subjects, defined as F = 2PR/(P + R). Here, P and R represent precision and recall respectively. Precision reflects the probability that the detected edge is valid, and recall denotes the probability that the ground truth edge is detected.

### 4.1. Analysis of V1 CRF Characteristics

**Figure 4:**
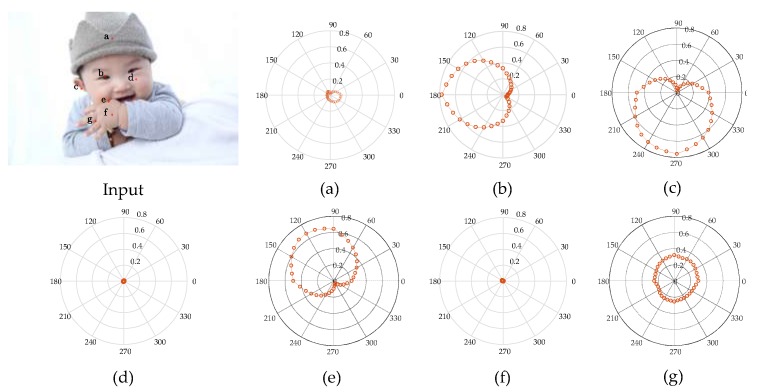
Analysis of the CRF response characteristics of V1 complex cells. (**a**–**g**) correspond to the responses of the pixel points at positions a–g in the Input, respectively.

To verify that the hierarchical contour detection model constructed in this paper can better simulate the feature perception and expression capacities of the cells at early visual stages and can better fit the biological characteristics, in connection with the more complex structures of a natural human image as shown in Figure 4, the CRF response characteristics of V1 complex cells for different areas of the image are analysed in specific experiments, and the results are as shown in Figure 4a–g, which correspond to the responses of the pixel points at positions *a*–*g* in the human image, and the polar coordinate values correspond to a group of 36 V1-cell model response values in different preferred directions. It can be seen in Figure 4 that the CRF of V1 cells is not simply an isotropic concentric circular structure but has obvious orientation selectivity, and this characteristic is most strongly manifested when the direction of the visual stimuli and the preferred direction of the cell are consistent; however, it shows a gradually decreasing trend when the response characteristics are in other directions. This kind of CRF characteristic is an important foundation for V1 cells to have the detection capability for image edges and contours. In border areas with greater changes in colour and luminance, the response of V1 cells consistent in direction with the visual stimuli has obvious orientation characteristics. For example, in connection with pixel point *b* at the edges of the human eye, cells with a preferred direction of 180° have the highest response value of 0.77 (as shown in Figure 4b); in connection with pixel point *c* at the boundary of the auricle, cells with a preferred direction of 270° have the maximal response value of 0.76 (as shown in Figure 4c); in connection with pixel point *e* at the edges of the hand, the greatest response value is 0.68 for cells with a preferred direction of 120° (as shown in Figure 4e), and so on. In areas with smaller changes in colour and luminance—that is, the internal area of the contour—all response values for direction-sensitive V1 cells are very small, such as pixel point *a* inside the brim of the hat in Figure 4a, pixel point *d* inside the cheek in Figure 4d, and pixel point *f* inside the hand in Figure 4f. In areas with disorderly changes in colour and luminance, all direction-sensitive V1 cells have a certain response, but the distinction in response value with difference preferred directions is small. In this case, it is difficult to differentiate the direction of the edge; for example, the response value of pixel point *g* between the fingers in Figure 4g is between 0.22 and 0.31.

**Figure 5:**
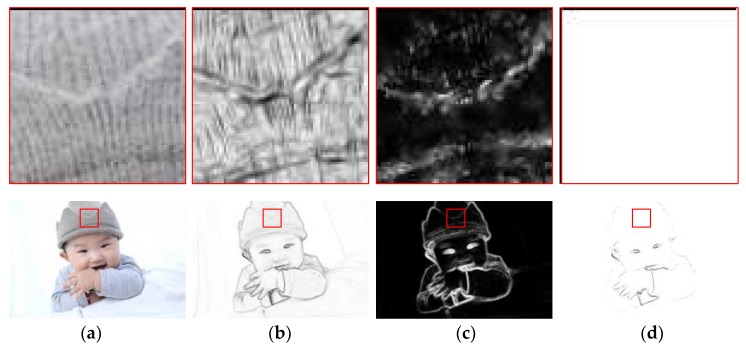
Demonstration of NCRF surround inhibitory effects. (**a**) Input, (**b**) V1 CRF response, (**c**) Modulation weight, (**d**) V1 output.

The characteristics of V1 CRF are analysed in Figure 5, but the V1-cell receptive field has unique structure; that is, the receptive field consists of sensitivity-oriented CRF and selective-suppression-oriented NCRF.

Therefore, to further understand the overall characteristics of the V1-cell receptive field, we analyse the surround inhibition effect of the receptive field when the V1 cells are processing natural images. As shown in Figure 5, the second row represents some of the response results in the contour detection of a complex human image with dimensions of 456 × 684, and the first row comprises locally enlarged 70 × 70 image blocks corresponding to the boxes marked in red. For the complex natural input image (Figure 5a), there are many disorderly internal textures in the response of V1 cells calculated by our CRF model. Without considering the NCRF surround inhibition, some wanted edges are embedded in these disorderly textures or cluttered backgrounds, as shown in Figure 5b. After the introduction of surround inhibition under the modulation of a variety of visual features, the strength of surround inhibition at every pixel position in the natural image is as shown in Figure 5c. The strength of surround inhibition is obviously stronger where there is intensely disorderly texture, such as the internal area of the brim of the hat, the internal area of the sleeve, and the blanket area; the suppression effect is obviously weaker in important contour areas, such as the face, the edges of the hand, and the edges of the hat. The V1 response outputs in Figure 5d show that the suppression model under multi-feature modulation proposed in this paper can effectively suppress disorderly texture and cluttered background while maintaining the integrity of salient contours.

### 4.2. Experiments on BSDS300/500 Dataset

**Figure 6:**
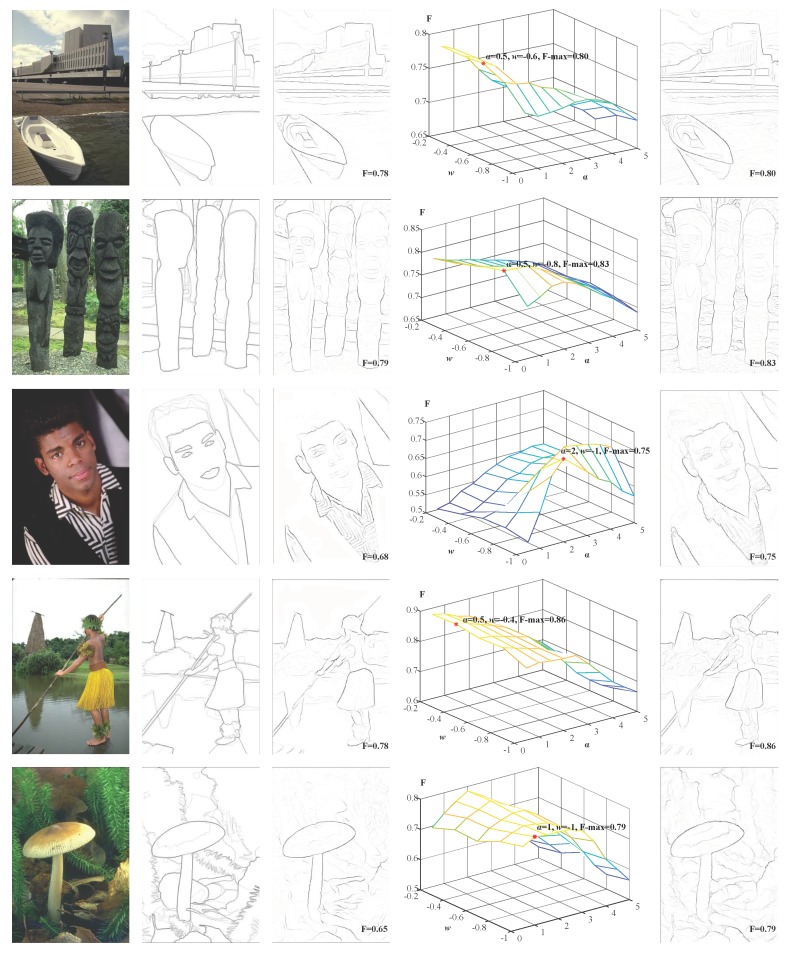
Results of BIHCD model with different parameters on BSDS300 samples. (**a**) Input, (**b**) Human, (**c**) Pb, (**d**) F-measure with various parameters, (**e**) *BIHCD.*

Natural images are complex and varied. To verify the actual performance of the BIHCD model in this paper, we selected the standard image database (Berkeley Segmentation Data Set 300/500 (BSDS300/500)) provided by the University of California, Berkeley, to test the performance of image contour detection in complex natural scenes. BSDS300 datasets contains 300 natural images (200 training and 100 test images) and BSDS500 additionally adds 200 test images. Each image provides 5–10 human-marked standard contour (Human) data, which lead to the presence of greater artificial subjective factors in the contours marked in the Human map, as shown in Figure 6. For example, in the mushroom sample in the last row of Figure 6, cluttered background such as weeds are marked as contours. Therefore, in subsequent experiments, we will average all Human map of the same image as its ‘Ground Truth’. In combination with the Human map, we calculated the aforementioned evaluation index (F-measure) in the experiment and drew a diagram of the corresponding precision-recall (P-R) curve, which directly reflected the consistency between the contours detected and obtained by different algorithms and the Human map.

First, we tested the two important parameters that affect our BIHCD model on BSDS300/500 datasets—*w* and *α*—and other parameters have been set in earlier text. To ensure the fairness and reasonableness of the performance comparison, we used only 200 training samples in the parameter selection process; in the test experiments, our BIHCD model was carried out only in connection with the test samples. After the experimental tests, the optimal parameter values were *w* = −0.8 and *α* = 1.0. In the following step, these parameter settings were fixed in different scenes when the proposed method is compared to other models. Figure 6 is a comparison of the contour detection results from the *BIHCD* algorithm of this paper and those from the classical Pb algorithm [6], where Figure 6a is the input image; Figure 6b is the averaged ‘Ground Truth’; Figure 6c is the contours detected and obtained by the *Pb* algorithm; and the corresponding F-measures are shown in the lower right corner. Figure 6d is a comparison of the F-measures obtained by calculating the contour detection results with different parameters, and Figure 6e is the optimal results of our BIHCD model corresponding to the maximal F-measure. Figure 6d shows that there are comparatively large gaps in visual information, such as luminance and colour, in the different sample images, and the connection weight w and the texture suppression strength α corresponding to the maximal F-value are also different. A comparison of Figure 6c with Figure 6e demonstrates that the BIHCD algorithm has better performance in detecting salient contour than the typical learning method Pb.

We then carried out objective comparisons between our BIHCD model and some other state-of-the-art biologically-inspired or machine-learning contour detection algorithms as shown in Table 1. Human refers to the mean value of the human contour annotation performance. Compared with the bottom-up algorithms based on low-level image features to which the model of *Canny* et al. belongs, the *BIHCD* algorithm of this paper uses low-level visual cues in the same way; therefore, it has characteristics such as fast speed and database-independent detection performance.

In contrast to the Canny algorithm, which uses only a single low-level image feature, our model uses the colour opponency characteristics of early vision to enhance the detection of colour boundaries and luminance boundaries while simultaneously combining with the surround inhibition effect under the modulation of multiple features, such as colour, distance, and direction, to effectively suppress the unwanted texture inside the image contours. Therefore, the contour detection indices—F-measure and AP-value—are greatly improved.

Models based on machine learning include the shallow learning models represented by Pb et al. and the deep learning models represented by holistically-nested edge detection (HED) [9] et al. Like the Pb algorithm, our model is an approach based on local strategy; that is, it uses only the image local multi-feature information to detect and locate the contours and has better detection performance. Compared with the characteristics of algorithms such as gPb [1] and HED, these models based on machine learning aim at operating on image blocks one by one but operates on the entire image, and the introduction of global features facilitates the acquisition of high-level information, which allows for better detection results to be obtained than our model. However, these methods are particularly time consuming because of many unknown parameters needed optimizing and the complex texture calculations and globalization with spectral clustering in both training and testing stages, especially deep learning algorithms. The mean computation time to compute one contour map with *gPb* is 133 s and *HED* is 213 s while our algorithms only takes 15 s with MATLAB R2017a (the computer used here is an Intel Core 2 CPU, 2.5 GHZ with 8.0 G RAM). At the same time, the detection performance of these models is directly related to the completeness of the training sample data. However, there are only two parameters that require optimizing in our model, and the robustness of algorithm to parameters is also higher. Therefore, this result indicated that our model yields a good trade-off between performance and complexity.

**Figure 7:**
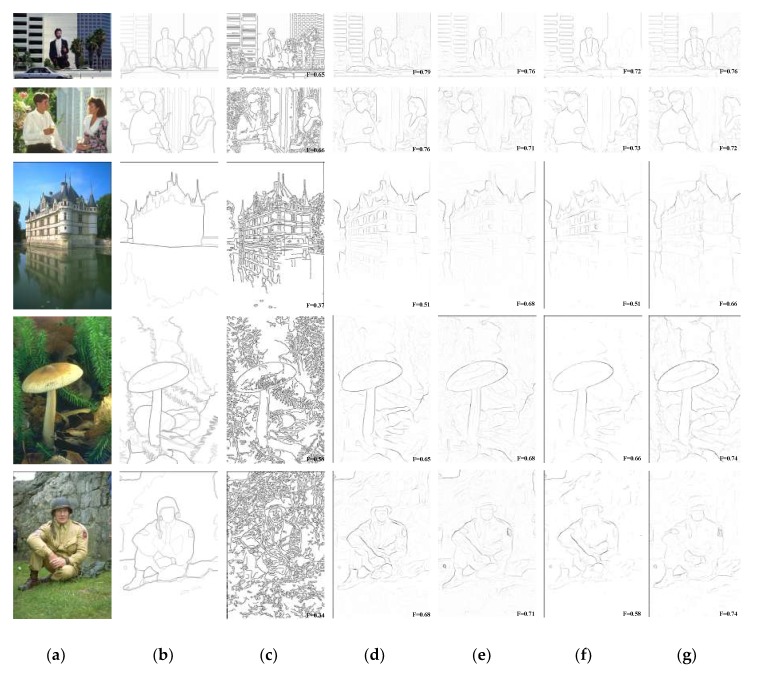
Results of various models on BSDS500 samples. (**a**) Input, (**b**) Human, (**c**) Canny, (**d**) Pb, (**e**) SCO (−0.7), (**f**) MCI, (**g**) BIHCD.

Figure 7 represents a comparison of the contour detection results on some of the images in the BSDS300/500 database by our model and by the typical state-of-the-art contour detection algorithms in Table 1. Note that the contours detected by *Canny* are the post-binarization results, and the other several algorithms and the Human map are the non-binarization results after the suppression of non-maximal values, which is mainly used to avoid the effect that the binarization of the candidate edge pixel ratio, parameter *p*, has on the detection performance of different algorithms. There are many unwanted texture edges inside the contours in the edges detected by Canny, which are interference information for the contours of the main structure of the image; therefore, compared with our BIHCD algorithm, the non-contour pixels detected in error are more numerous, and the evaluation index, the F-measure, is comparatively poor (one can see this by comparing Figure 7c,g). Although the Pb algorithm achieves contour detection through a machine learning method to comprehensively use the brightness gradient (BG), the colour gradient (CG), and the texture gradient (TG), it is more effective in detecting boundaries with low brightness contrast or low colour contrast (for example, the edges of the grey clothing in the upper right corner of the image with the soldier or the colour boundaries inside the mushroom, etc.) due to the colour opponency characteristics of early visual stages introduced in this paper. In addition, the NCRF surround modulation with multi-feature cues also ensures that, compared with the Pb algorithm, our method has better results in suppressing the cluttered textures inside the contours (for example, the details and edges of the windows inside the building). On the other hand, our model shows greater robustness in textural areas and better performance at detecting continuous lines, compared to the colour opponency-based *SCO* algorithm [31], which only used the spatial sparsity of the edge response to carry out texture suppression and ignored the effects of the regional colour and texture features in the image on internal texture suppression (one can see this by comparing Figure 7e,g). Thanks to the NCRF surround modulation, it is apparent that our BIHCD model is successfully suppresses the textural information originating from the background straws, as shown in the last two rows of Figure 7. At the same time, the MCI model [10] uses only the luminance information of the image to detect contours, and it belongs to the single-scale texture suppression method; therefore, the contour detection results in places such as the colour boundaries (for example, the mushroom and the surrounding green weeds) is poorer than that of our algorithm.

**Figure 8:**
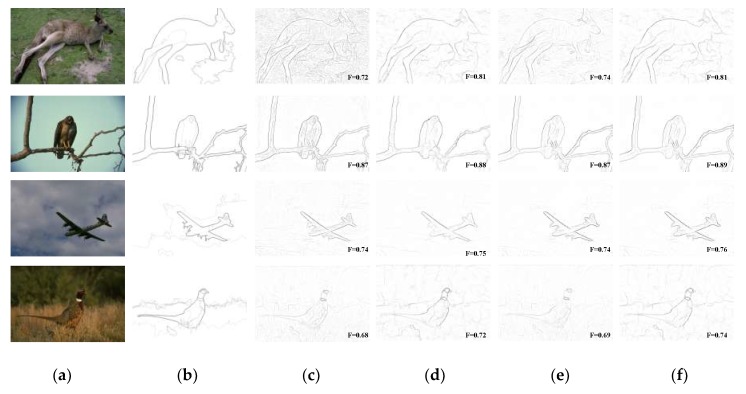
Evaluations of every component of our BIHCD model. (**a**) Input, (**b**) Human, (**c**) No V2 stage, (**d**) No V2 feedback, (**e**) No multiple, (**f**) BIHCD model.

In addition, by viewing Figure 7 and Figure 8 comprehensively, compared with SCO and MCI, which are both state-of-the-art biologically-inspired models proposed within last few years, our BIHCD model introduced the contour integration process of the V2 cells, explored the neural connections and pooling mechanism between visual areas with new physiological findings and proposed the information feedback connection between regional cells, which better fit the biological visual cognition mechanism and therefore obtained better objective and subjective contour detection results. Comparison of “No V2 stage” and “No V2 feedback” results in Figure 8 reveals that the V2 module strongly assist the process of eliminating textural and noisy patches. This is because that perception of shape extracted in V2 is significantly influenced by points where multiple edges meet, e.g., corners. From Figure 8d,f, it is obvious that shape feedback reinforces the desired contours by accounting for the impact of global shapes feedback sent from area V2 to V1 on local contours, e.g., the luminance of airplane contours is higher in the latter one. This is in line with previously findings and experimental results in [32,42] that feedback connections may amplify the features extracted in V1. Comparing “BIHCD model” with “No V2 feedback” results, it can tell us that multi-feature suppression and integration method under the guidance of multi-scale information helps constantly suppress texture boundaries, enhance contour of small target and also keep contour of large target, e.g., the cluttered textures and inner boundaries in the deer.

**Figure 9:**
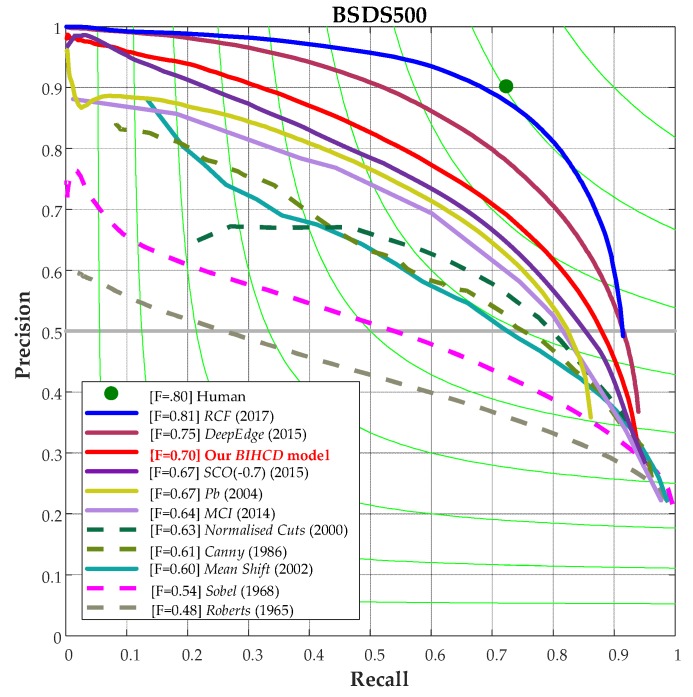
An overall performance comparison of the P-R curve on BSDS500.

Finally, Figure 9 shows a comparison of the P-R curves for the BSDS500 image database from our BIHCD model and other contour detection models. It can be seen in the figure that our algorithm attains a higher recall ratio and accuracy rate on the P-R curve than the classical Canny and Pb algorithms and the SCO and MCI models based on the biological vision mechanism, better illustrating that our algorithm simultaneously has better results in edge detection and texture suppression.

## 5. Conclusions

In this paper, we drew lessons from the perceptual characteristics of the cells at early visual stages for features such as edges, shapes, and colours and proposed a hierarchical contour extraction method based on the human vision mechanisms. Our key contributions can be summarized as follows: (i) exploring the receptive field computational models of various cells from retina to V2 and proposing a new biologically-inspired framework for image contour detection; (ii) analyzing and extending current neural models for contour detecting by accounting for feedback connections and pooling mechanism between V1 and V2; (iii) introducing a center-surround mechanism based model by combining multiple local cues; (iv) accounting for the property that salient and meaningful contours are more likely to be retained at different scales by integrating the information at multiple scales. We quantitatively compared our model to current state-of-the-art algorithms on both synthetic images and benchmark dataset, and our results show a significant improvement compared to the other biologically-inspired models while being competitive to the machine learning ones. Experimental results also demonstrate that our model achieves a good trade-off between edge detection and texture suppression.

This paper mainly simulated the hierarchical perception characteristics of different brain regions in capturing the image contours, which is mainly still based on the processing of low-level, local visual information. Actually, the visual cells have comparatively strong dynamic perception characteristics, and higher-level cells have a special information feedback effect on low-level cells (such as V4). Therefore, our future work will be based on the study of a rapid contour extraction method driven by the target information and global features in complex visual scenes, and we will integrate a higher level of a variety of visual features to improve contour detection performance and achieve other computer vision tasks.

## Figures and Tables

**Table 1:** Results of several contour detection algorithms on BSDS300/500.

Method			F(BSDS300)	AP(BSDS300)	F(BSDS500)	AP(BSDS300)
		Human	0.79	-	0.80	-
Machine learning	Shallow	*Pb*	0.63	-	0.67	-
		*BEL*	0.65	-	0.61	-
	Deep	*gPb*	0.70	0.66	0.71	0.65
		*Deep Edge*	-	-	0.75	0.80
		*HED*	-	-	0.78	0.83
Low-level features	Classical edge detection	*Canny*	0.58	0.58	0.61	0.58
		*Normalised Cuts*	0.62	0.42	0.63	0.45
	Biological	*SCO*	0.66	0.70	0.67	0.71
		*MCI*	0.62	-	0.64	-
		Ours	0.68	0.70	0.70	0.74
